# Systemic Sclerosis Complicated by Rapidly Progressive Osteomyelitis: A Case Report

**DOI:** 10.2174/0115733971365099250205151000

**Published:** 2025-02-06

**Authors:** Angelo Nigro

**Affiliations:** 1 Department of Rheumatology of Lucania, UOSD of Rheumatology, “Madonna delle Grazie” Hospital, Matera, Italy

**Keywords:** Systemic sclerosis, osteomyelitis, radiographic monitoring, antibiotic therapy, acrocyanosis, osteolysis, bone damage

## Abstract

**Background:**

Systemic sclerosis (SSc) is an autoimmune disorder characterized by progressive fibrosis and vascular complications. Osteomyelitis is a rare but serious complication in patients with systemic sclerosis, particularly those with advanced vascular compromise. This case is notable for the rapid progression of osteomyelitis and highlights the importance of early intervention and thorough clinical monitoring.

**Case Presentation:**

We report the case of a 68-year-old female with SSc (Scl-70 positive), treated with iloprost IV, nifedipine, bosentan, prednisone, and mycophenolate for pulmonary involvement. In January 2024, she developed acrocyanosis and severe pain in the fifth toe of the right foot. A small ulcer formed, and subsequent radiographic evaluation revealed rapid progression of osteolysis. Despite negative culture swabs, an infectious process was suspected, and combination antibiotic therapy was initiated. This treatment led to a gradual resolution of symptoms, with subsequent imaging showing detachment of the fifth toe.

**Conclusion:**

This case highlights the critical need for vigilant radiographic monitoring and timely antibiotic intervention in patients with SSc who develop vascular complications. Early diagnosis and treatment are crucial for optimizing patient outcomes and preventing severe bone damage.

## 1 INTRODUCTION

Systemic sclerosis (SSc) is an autoimmune disorder characterized by progressive fibrosis of the skin and internal organs, accompanied by a spectrum of vascular and osteoarticular complications [1, 2]. Among these, osteomyelitis represents a rare yet potentially severe complication, particularly in individuals with advanced vascular compromise [3, 4]. The rationale for reporting this case lies in the rapid progression of osteomyelitis despite early treatment of acrocyanosis, demonstrating the importance of continuous radiographic monitoring and timely intervention, which is not well-documented in previous literature. This report details a case of rapidly progressive osteomyelitis in a patient with SSc, emphasizing the importance of close radiographic monitoring and prompt diagnostic measures to ensure effective clinical management.

## 2 CASE REPORT

**Fig. (1):**
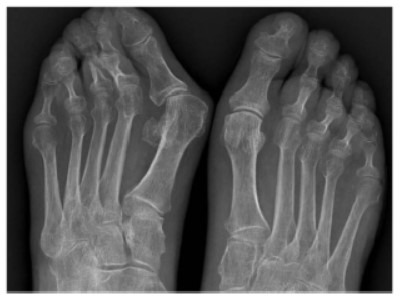
Initial radiograph of the feet, showing no abnormalities in the fifth toe of the right foot. Taken on January, 2024.

A 68-year-old female patient with a diagnosis of SSc (Scl-70 positive) was undergoing treatment with iloprost IV, nifedipine 30 mg daily, bosentan, prednisone 5 mg daily, and mycophenolate 2 g daily for interstitial lung disease. In January 2024, the patient presented with pain and acrocyanosis in the fifth toe of the right foot, which was associated with significant pain and difficulty in weight-bearing. An initial radiograph of the feet (Fig. **1**) was performed, which showed no abnormalities. After the onset of cyanosis, a small ulcer developed, exacerbating the pain, particularly during weight-bearing. Iloprost infusions were administered for six days daily, resulting in improvement in cyanosis but without significant pain relief.

**Fig. (2):**
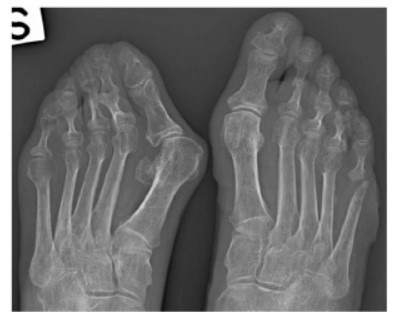
Follow-up radiograph three months later, showing clear signs of osteolysis in the fifth toe of the right foot. Taken on March, 2024.

Three months later, the patient developed erythema and swelling in the right foot, predominantly affecting the fifth toe. A follow-up radiograph (Fig. **2**) demonstrated clear signs of osteolysis, highlighting the importance of timely radiographic surveillance for detecting the rapid progression of bone damage in patients with SSc.

During the clinical course, two culture swabs were obtained from the ulcer, both yielding negative results despite the presence of purulent drainage mixed with blood. This suggested osteomyelitis of unknown etiology. The patient was subsequently started on combination antibiotic therapy, including ceftriaxone 2 g daily and clindamycin 600 mg three times daily, which led to gradual resolution of symptoms and reduction in pain.

**Fig. (3):**
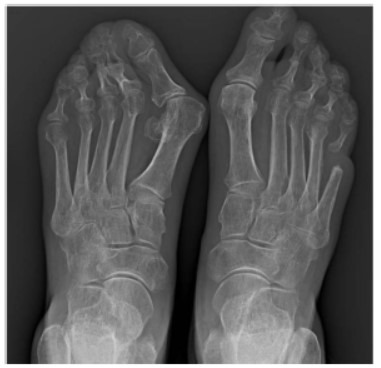
Radiographic evaluation nine months after the initial presentation, demonstrating detachment of the two stumps of the fifth ray of the right foot. Taken on September, 2024.

A subsequent radiographic evaluation was performed nine months later (Fig. **3**), which demonstrated detachment of the two stumps of the fifth ray of the right foot. This case highlights how SSc can predispose patients to rapid progression of osteomyelitis, underscoring the value of frequent radiographic assessments for early detection. Timely intervention with antibiotic therapy was crucial in managing the rapid evolution of bone lesions.

## 3 DISCUSSION

Systemic sclerosis is a complex disorder that predisposes patients to various vascular and osteoarticular complications [5, 6], including osteomyelitis. In the present case, the patient exhibited rapid osteolysis of the fifth toe of the right foot, identified through a radiographic examination conducted four months after the initial presentation [7, 8]. The sequence of acrocyanosis, followed by a painful ulcer and subsequent radiographic evidence of osteolysis, indicated an underlying bone infection despite negative microbiological findings. Prompt initiation of antibiotic therapy resulted in significant clinical improvement, emphasizing the necessity for early diagnosis of infectious processes to mitigate complications [9, 10].

Compared to previously reported cases, this case highlights the unusually rapid progression of osteomyelitis in the context of SSc despite aggressive vascular management and the absence of positive microbiological cultures. The combination of iloprost, antibiotics, and close radiographic follow-up provided a unique approach to management, emphasizing the need for multidisciplinary care in patients with severe vascular involvement. Management of osteoarticular complications in patients with SSc needs a multidisciplinary approach and diligent follow-up [11, 12]. Specifically, radiographic surveillance was pivotal in this case for the early identification of osteomyelitis, allowing timely therapeutic intervention [13]. This report underscores the importance of considering osteomyelitis as a differential diagnosis in patients with SSc who present with persistent painful lesions, even in the absence of positive microbiological evidence [14, 15].

## CONCLUSION

This case report illustrates a rare but severe complication of SSc, a rapidly progressive osteomyelitis effectively managed with antibiotic therapy. Early identification of the infectious process, if possible, and timely intervention are critical to achieving favorable clinical outcomes. Regular radiographic monitoring and early diagnosis are fundamental to optimizing therapeutic management and preventing further complications in patients with SSc.

## AUTHORS’ CONTRIBUTIONS

The author confirms sole responsibility for the following: study conception and design, data collection, analysis and interpretation of results, and manuscript preparation.

## Data Availability

All the data and supporting information are provided within the article.

## ABBREVIATION

SSc: Systemic Sclerosis

## STANDARDS OF REPORTING

CARE guidelines were followed.
